# Effect of Female Genital Schistosomiasis and Anti-Schistosomal Treatment on Monocytes, CD4^+^ T-Cells and CCR5 Expression in the Female Genital Tract

**DOI:** 10.1371/journal.pone.0098593

**Published:** 2014-06-04

**Authors:** Elisabeth Kleppa, Veron Ramsuran, Siphosenkosi Zulu, Gunn Hege Karlsen, Alfred Bere, Jo-Ann S. Passmore, Patricia Ndhlovu, Kristine Lillebø, Sigve D. Holmen, Mathias Onsrud, Svein Gunnar Gundersen, Myra Taylor, Eyrun F. Kjetland, Thumbi Ndung’u

**Affiliations:** 1 Norwegian Centre for Imported and Tropical Diseases, Department of Infectious Diseases, Oslo University Hospital (OUH), Oslo, Norway; 2 Faculty of Medicine, University of Oslo, Oslo, Norway; 3 HIV Pathogenesis Programme, Nelson R Mandela School of Medicine, University of KwaZulu-Natal (UKZN), Durban, South Africa; 4 School of Public Health Medicine, Nelson R Mandela School of Medicine, UKZN, Durban, South Africa; 5 Aarhus University, Aarhus, Denmark; 6 Emory Vaccine Center, Emory University, Atlanta, Georgia, United States of America; 7 Division of Medical Virology, IDM, University of Cape Town, Cape Town, South Africa; 8 Imperial College, London, United Kingdom; 9 Department of Gynaecology, OUH, Oslo, Norway; 10 Research Unit, Sorlandet Hospital, Kristiansand, Norway; 11 Centre for Development Studies, University of Agder, Kristiansand, Norway; Institut de Recherche pour le Développement, France

## Abstract

**Background:**

*Schistosoma haematobium* is a waterborne parasite that may cause female genital schistosomiasis (FGS), characterized by genital mucosal lesions. There is clinical and epidemiological evidence for a relationship between FGS and HIV. We investigated the impact of FGS on HIV target cell density and expression of the HIV co-receptor CCR5 in blood and cervical cytobrush samples. Furthermore we evaluated the effect of anti-schistosomal treatment on these cell populations.

**Design:**

The study followed a case-control design with post treatment follow-up, nested in an on-going field study on FGS.

**Methods:**

Blood and cervical cytobrush samples were collected from FGS negative and positive women for flow cytometry analyses. Urine samples were investigated for schistosome ova by microscopy and polymerase chain reaction (PCR).

**Results:**

FGS was associated with a higher frequency of CD14^+^ cells (monocytes) in blood (11.5% in FGS+ vs. 2.2% in FGS-, p = 0.042). Frequencies of CD4^+^ cells expressing CCR5 were higher in blood samples from FGS+ than from FGS- women (4.7% vs. 1.5%, p = 0.018). The CD14^+^ cell population decreased significantly in both compartments after anti-schistosomal treatment (p = 0.043). Although the frequency of CD4+ cells did not change after treatment, frequencies of CCR5 expression by CD4+ cells decreased significantly in both compartments (from 3.4% to 0.5% in blood, p = 0.036; and from 42.4% to 5.6% in genital samples, p = 0.025).

**Conclusions:**

The results support the hypothesis that FGS may increase the risk of HIV acquisition, not only through damage of the mucosal epithelial barrier, but also by affecting HIV target cell populations, and that anti-schistosomal treatment can modify this.

## Introduction

Heterosexual intercourse is the most frequent mode of transmitting Human Immunodeficiency Virus (HIV), infecting women by entry into HIV target cells in the genital mucosa [1], [2]. Sexually transmitted infections (STIs) have been hypothesized to increase HIV susceptibility by causing breaches in the genital epithelium, by increasing the number of HIV target cells and by immune activation in the genital mucosa [3], [4]. The mechanisms of HIV transmission to target cells and the immune responses in the genital tract are still poorly understood [5]. Monocytes and T-cells expressing the CD4 receptor and the chemokine receptor CCR5 can be infected with HIV [6]–[8]. Factors that influence the number of HIV target cells and their receptors may increase the risk of HIV transmission at the site of exposure [4], [9]–[12]. Recently, several studies have found that women from sub-Saharan Africa have increased systemic and genital immune activation compared to women from Europe and North America [13]–[16]. It has been hypothesized that this may be related to regional diseases such as schistosomiasis [13]–[16].

**Figure 1:**
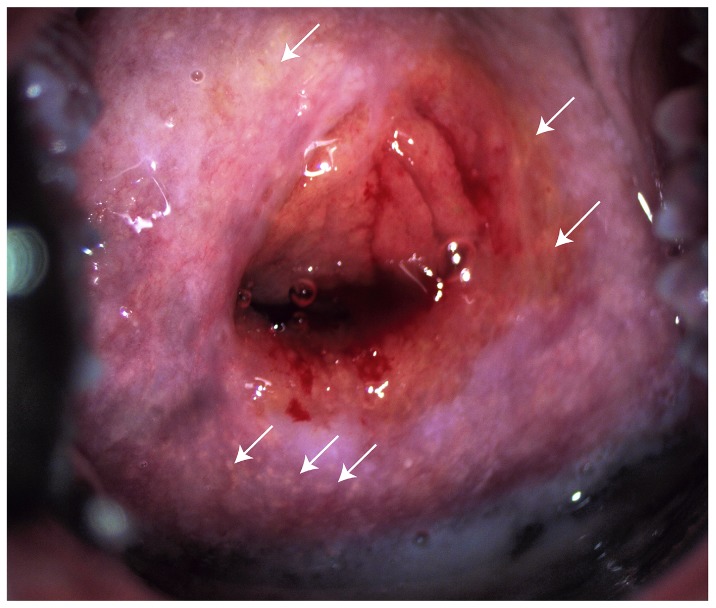
Colposcopic image of cervix with female genital schistosomiasis. The image shows sandy patches appearing as grains (arrows point to some examples) and contact bleeding.

*Schistosoma (S.) haematobium* is a poverty-related, waterborne parasite which coexists with HIV in many countries [15]. Of the parasitic diseases, only malaria has a larger socioeconomic and public health impact than schistosomiasis [17]. Women with schistosomiasis may have lesions in the genital tract, a condition referred to as female genital schistosomiasis (FGS, Figure 1), which has been hypothesized to be a co-factor for HIV transmission similar to STIs [18]. Histopathological studies have demonstrated that the tissue surrounding the schistosomal ova is more densely vascularized and have more CD4^+^ T-cells than healthy cervical tissue [19], [20]. Cross-sectional studies have found a three- to four-fold increased odds of having HIV in women with urogenital schistosomiasis [21], [22]. A recent study showed a similar tendency in individuals infected with *S. mansoni* [23]. Furthermore, the intestinal mucosa of macaques with *S. mansoni* infection has been shown to be 17-fold more susceptible to HIV infection than healthy controls [24], [25]. Praziquantel is the recommended treatment for schistosomiasis, however, the effect on genital lesions is still unknown [26].

In humans, little is known about the effect of genital parasite infections on the susceptibility to HIV [13]. To our knowledge, no studies have explored the presence of HIV receptors and co-receptors in the genital and blood compartments in young women with FGS. In this in-vivo study, we investigated the influence of FGS on HIV target cells in blood and in the genital compartment of women.

## Methods

### Enrolment and sampling

The women were part of a cohort of high-school students included in a prospective intervention study in rural KwaZulu-Natal, South Africa. The area is endemic for *S. haematobium* and the infection is prevalent from an early age [27]. Frequent contact with fresh water bodies is common in the area, both for recreational purposes and chores such as laundry [28]. *S. mansoni* is not endemic in the district (in a subsample of the main cohort, no *S. mansoni* ova were found in 853 stool samples. One sample per person was examined microscopically by the Kato-Katz method, unpublished).

We recruited FGS+ and FGS- young females in a nested case-control study. Sexually active females from the highest grades of high school were included. The samples were collected from May to June 2011 and January to February 2012. Baseline enrolment was done in two rounds with an interval of approximately 8 months. In total, 14 FGS+ women were enrolled in the first round and post-treatment samples collected in January and February 2012. All visits followed the same protocol. Women with FGS were treated with praziquantel (40 mg/kg) at baseline.

**Figure 2:**
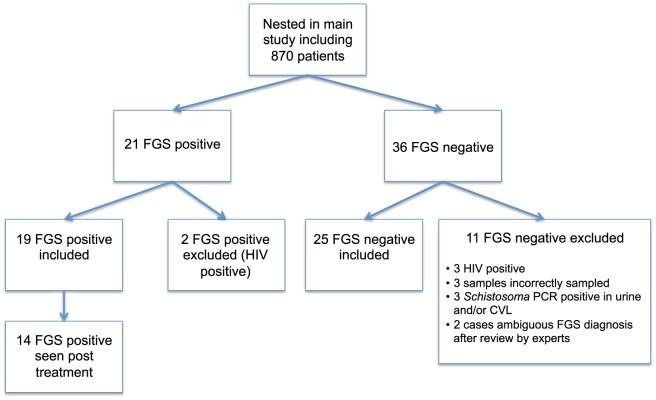
Flowchart. Flowchart showing the inclusion of study participants (FGS =  female genital schistosomiasis, CVL =  cervicovaginal lavage).

FGS+ cases were included based exclusively on findings of pathognomonic sandy patches by photocolposcopic investigation [18] (Figure 1).Women were excluded if they reported having HIV, were menstruating or had visible signs or symptoms of an STI. For comparison, control samples were collected consecutively from women enrolled in the main study without sandy patches and with no finding of schistosome eggs by microscopy of urine. PCR detection of schistosome DNA was run on urine and cervicovaginal lavage (CVL), and clinically negative controls who had a positive PCR result were excluded (Figure 2). Women who were found to be HIV seropositive, or had cervical sampling incorrectly done, were excluded.

### Ethics

Research ethical committees in South Africa and Norway approved the study (Biomedical Research Ethics Committee (BREC), University of KwaZulu-Natal (Ref BF029/07), KwaZulu-Natal Department of Health, (Reference HRKM010-08) and the Regional Committee for Medical and Health Research Ethics (REC), South Eastern Norway (Ref 469-07066a1.2007.535)). Furthermore, the Departments of Health and Education in Ugu District, KwaZulu-Natal gave permissions for this study. All participants signed individual, written informed consents. The ethical committees, BREC and REC, were aware that 16 and 17 year olds were participating in the study and specifically approved the consent procedure (independent minor consent, no parental consent). According to South African legislation, persons over the age of 12 may consent independently to participate in research. STI treatment was offered to participants with clinical signs and symptoms, and their partners in accordance with the South African syndromic treatment protocol [29]. They were also treated at follow-up if the woman tested positive on laboratory analyses. Anti-schistosomal treatment was offered to all as part of a mass drug administration campaign. Voluntary HIV testing and follow-up was done in accordance with South African guidelines with pre- and post-test counseling. HIV+ women were referred to local clinics for follow-up and treatment. South Africa provides universal access to antiretroviral treatment.

### Clinical investigation and specimen collection

The gynecological examinations were performed by two trained female medical doctors (EK, KL). All cases had extensive, characteristic sandy patches with deep and/or superficial grains visible in the cervical mucosa. Colposcopic examination was done of the entire cervicovaginal surface (Olympus OCS 500 Photocolposcope with a mounted Olympus E420 10 megapixels (Mpx) or a Leisegang colposcope with a Canon EOS 40D 10 Mpx). For quality control purposes, lesion sizes and localization were documented. Quality control was done by clinicians with extensive experience in FGS and gynecology (EFK, MO).

The investigation commenced by collecting cervical cells using an endocervical cytobrush (EndoCervex-brush, Rovers, Oss, The Netherlands) as previously described [30]–[33]. Briefly, the brush was rotated 360° in the cervical os. After collection, the cytobrush samples were placed in a transport medium (R10 made of RPMI supplemented with 10% foetal calf serum (FCS), HEPES buffer, L-glutamine, streptomycin, and penicillin) at room temperature for transport to the laboratory. CVL samples were then collected by flushing 10 mL of sterile saline on the cervical surface four times before drawing it back into a syringe and depositing it into tubes for frozen storage. Urine was collected from each woman, between 10 am and 2 pm. In addition, whole blood (30 mL) was collected into sterile acid-citrate-dextrose anti-coagulated Vacutainer tubes (Becton, Dickinson and Company (BD), Franklin Lakes, NJ, US). Blood and cervical cytobrush samples were transported to the laboratory within four hours of collection.

### STI and schistosomiasis testing

Baseline CVL samples were subsequently analyzed by strand displacement assay (SDA) for *Neisseria gonorrhea* (ProbeTec CT/GT, BD) and *Chlamydia trachomatis* (ProbeTec CT/GC, BD). PCR was used to detect *Trichomonas (T.) vaginalis* (in-house PCR, Laboratory of Infection, Prevention and Control (IPC), University of KwaZulu-Natal (UKZN), Durban, South Africa) and *Herpes simplex virus* (in-house PCR, IPC). Syphilis was detected in thawed serum samples using Macro Vue test 110/112 for Rapid plasma reagin (RPR), (BD), and Immutrep for *Treponema pallidum* hemagglutination assay (TPHA; Omega diagnostics Group PLC, Alva, Scotland, UK). HIV testing was done using Bioline Rapid Test HIV, (NJ, US) and confirmatory Sensa Tri-Line HIV Test Kit (Pantech, Durban, South Africa).

CVL and urine samples were analysed using an in-house real-time PCR technique for detection of schistosome DNA (Leiden University Medical Centre, Leiden, Netherlands)[34].

Microscopy was performed by adding merthiolate-formalin solution (2%) to ten mL of urine, centrifuging and depositing the whole pellet on one or more glass slides. Schistosome ova in urine were counted and a positive diagnosis was recorded if at least one ovum was seen.

### Flow cytometry on cervical cytobrush and peripheral blood mononuclear cells

In the laboratory, the cervical cytobrush samples were processed for genital tract cells and blood for peripheral blood mononuclear cells (PBMCs). The cytobrushes were flushed 30 times with R10 in the tube using a pipette. The suspension was then transferred to a new tube and centrifuged at 2100 rpm for 3 minutes. After a second washing, the pellet was resuspended in 2 mL phosphate buffered saline (PBS). In addition, fresh PBMCs were isolated using Ficoll-Hypaque density gradient centrifugation (Histopaque-1077, Sigma-Aldrich, St. Louis, MO, US). All of the cervical cytobrush cells and 2×10^6^ PBMCs were stained for 20 minutes in the dark, first with ViVid LIVE/DEAD (Invitrogen, NY, US), then with a panel including CD8 Qdot (clone: 3B5, Invitrogen, NY, US), CD3 APC H7 (clone: SK7), CD4 PerCP Cy 5.5 (clone: L200), CD56 PE Cy 7 (clone: B159), CD14-fluorescein isothiocyanate (clone: M5E2) and CCR5 APC (clone: 2D7/CCR5, all from BD, San Jose, CA, US). The samples were then washed with 2 mL of PBS, fixated using paraphormaldehyde (1%) and stored in the fridge over night before analysis next morning. Staff analyzing the samples were blinded to the FGS status.

**Figure 3:**
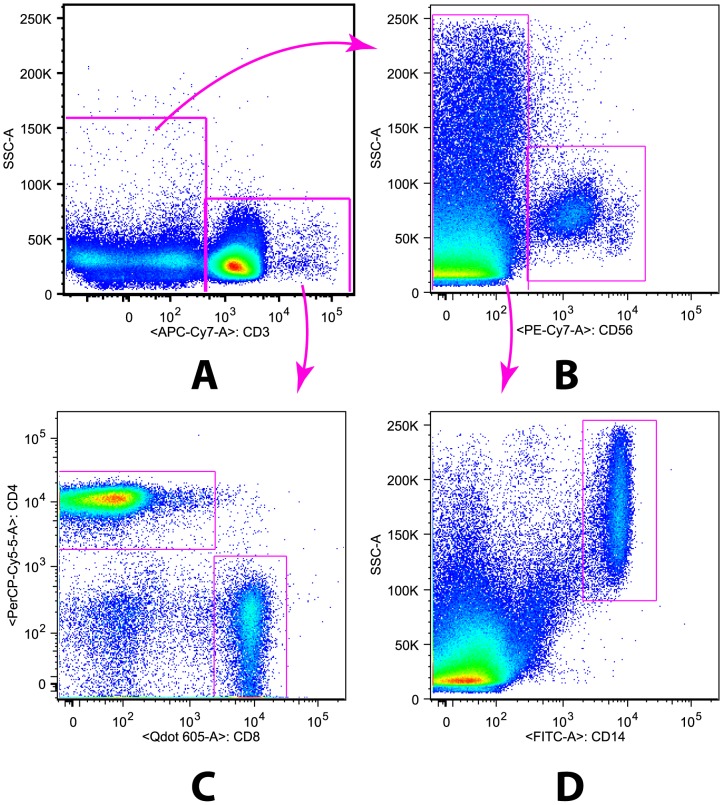
Gating strategy. Figures showing the gating strategy for A) CD3^+^ B) CD3^−^CD56^+^ C) CD3^+^CD4^+^ and CD3^+^CD8^+^ and D) CD3^−^CD56^−^CD14^+^.

Cells were acquired using an LSR II flow cytometer (BD) and FACSDiva software (BD). The data was analyzed using FlowJo software (Tree Star, Inc. version 9). Compensation and calibration was performed daily using BD CaliBRITE beads. CD4 lymphocytes were defined and gated as CD3^+^CD4^+^ cells (Figure 3C), while monocytes were defined and gated as CD3^−^CD56^−^CD14^+^cells (Figure 3D). CCR5 positive cells on monocytes, which express CD14, were defined and gated as CD3^−^CD56^−^CD14^+^CCR5^+^ cells. Percentages of cells expressing a given population were represented as a proportion of the parent population. The proportion of cells in each subpopulation of cells was compared in FGS positives and negatives. Back gating was used to confirm the identity of the cell populations.

### Statistical analyses

Statistical analyses were performed using IBM SPSS Statistics version 20 (Armonk, NY, US). The Mann-Whitney U test was used to compare the results from the flow cytometry in FGS+ and FGS- and confidence intervals are given as inter quartile ranges (IQR), as the data did not follow a normal distribution. To compare results from samples collected before and after treatment the Wilcoxon signed rank test was used. Pearson’s chi-square test or Fisher’s exact test were used to compare the groups for STIs. Samples yielding a minimum of 3,000 events after gating for CD3^+^ cells were included for further statistical analyses. A significance level of 5% was used.

## Results

We collected samples for flow cytometric analyses from 19 FGS+ and 25 FGS- women (Figure 2). Median age of the women in this study was 18 years (range 15–23, Table 1). Of the 44 participants included, 30 (68%) met the criterion of a minimum of 3,000 CD3 positive events in the flow cytometry analysis in both the mucosal and the blood sample. A median of 96,996 events (live, CD3^+^ cells) in blood and 7,626 (live, CD3^+^ cells) in cervical samples was analyzed.

**Table 1:** Characteristics of study participants by female genital schistosomiasis (FGS) status.

Variable	FGS positive (n = 19)^a^		FGS negative (n = 25)^a^		p^b^
	n	(%)	n	(%)	
Median age at baseline	18		17		0.030^c^
Schistosome ova in urine microscopy^d^	12	(63%)	0	(0%)	
Schistosoma PCR positive in urine^d^	12	(63%)	0/20		
Schistosoma PCR positive in vaginal lavage^d^^,^ e	5	(26%)	0/20		
*Trichomonas vaginalis*	8	(42%)	1/21	(5%)	0.007
*Herpes simplex* virus	1	(5%)	0/20	(0%)	0.490
*Neisseria gonorrhoea*	3/13	(23%)	0/17	(0%)	0.070
*Chlamydia trachomatis*	4/13	(31%)	2/17	(12%)	0.360
*Treponema pallidum*	0	(0%)	2/9	(22%)	0.095
Injectable contraceptive	7	(28%)	6	(32%)	0.800

a Analysis done on all if not stated otherwise.

b Pearson’s chi-square or Fisher’s exact test.

c Mann-Whitney U test.

d Not compared due to inclusion criteria of the negative group.

e The pathology in female genital schistosomiasis is also due to dead, calcified ova. PCR may therefore be negative.

A median of 4 STI analyses (range 0–5) were run in the patients depending on the availability of specimens. FGS was significantly associated with *T. vaginalis* (p = 0.007), but not with the other STIs (Table 1). The FGS negative group did not differ from the main study cohort when comparing STI prevalence (data not shown). In the FGS+ group, 12 (63%) women had *S. haematobium* ova in urine (Table 1). In the FGS+ women, there were similar levels of STIs in the group with *S. haematobium* ova in urine (microscopy) and the group with negative urines (p = 0.68). Of the FGS positive women, 14 out of the original 19 were seen 7–8 months after they had been given anti-schistosomal treatment. At follow-up, none of the treated cases had ova in urine (microscopy). One patient reported to have received STI treatment between baseline and follow-up and was therefore excluded from the analyses.

### HIV target cells in blood samples

**Figure 4:**
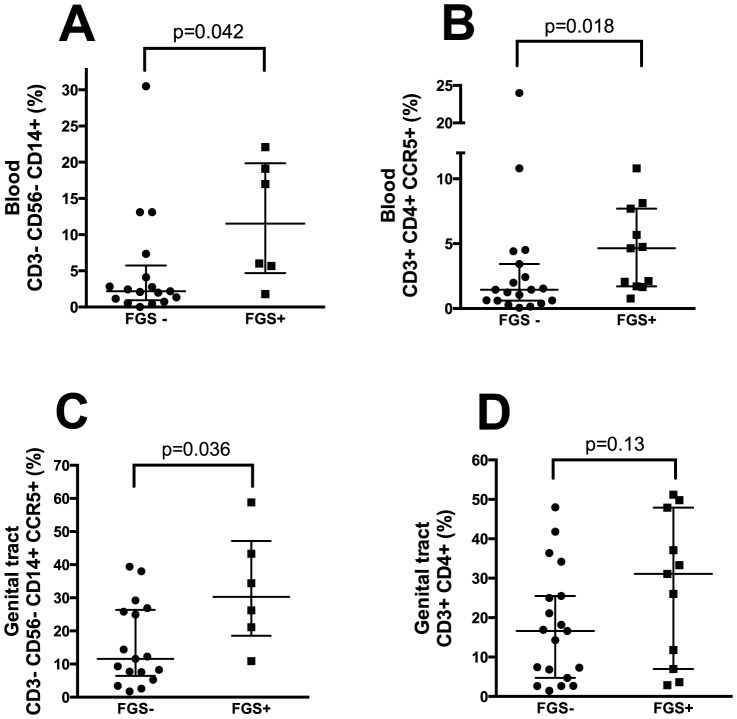
Comparison of FGS+ and FGS−. Figures comparing the FGS positive (genital sandy patches) and negative (no genital sandy patches, negative *Schistosoma* PCR in cervicovaginal lavage/urine and negative urine microscopy for ova). Figures show blood (A–B) and cervical samples (C–D).

Women with FGS had a significantly higher proportion of CD3^−^CD56^−^CD14^+^ cells than FGS- women in blood samples (median 11.5% (IQR 4.7–19.9) vs. 2.2% (IQR 0.9–5.7) respectively, p = 0.042; Figure 4A), whereas there was no significant difference for CCR5 expression on CD14^+^ cells (p = 0.89; data not shown). CD4^+^ T-cells expressing CCR5 were more frequent in FGS positives than negatives (median 4.7% (IQR 1.7–8.1) vs. 1.5% (IQR 0.6–3.4) respectively, p = 0.018; Figure 4B). However, the proportion of CD3^+^CD4^+^ T-cells was not significantly different in FGS negatives and positives (median 54% and 51% respectively, p = 0.86; data not shown).

### HIV target cells in genital samples

In cervical cytobrush derived cells, the proportion of CD3^−^CD56^−^CD14^+^ monocytes was not significantly different in FGS negatives and positives (median 1.13 in both groups, p = 0.62; data not shown). The CCR5 expression on CD14^+^ cells was higher in the FGS positive group than the FGS negative group (30.3% (IQR 18.6–47.2) vs. 11.6% (IQR 6.4–26.4) respectively, p = 0.036; Figure 4C). There was no significant difference in CD3^+^CD4^+^ T-cells in FGS+ individuals compared to FGS- individuals in the genital samples (median 16.6 (IQR 4.7–25.5) vs. 31.1 (IQR 7.0–47.9) respectively, p = 0.13; Figure 4D). No difference was found when comparing CD4^+^ T-cells expressing CCR5 in FGS+ and FGS- women (p = 0.29; data not shown).

### Effect of praziquantel treatment on HIV target cells in blood

**Figure 5:**
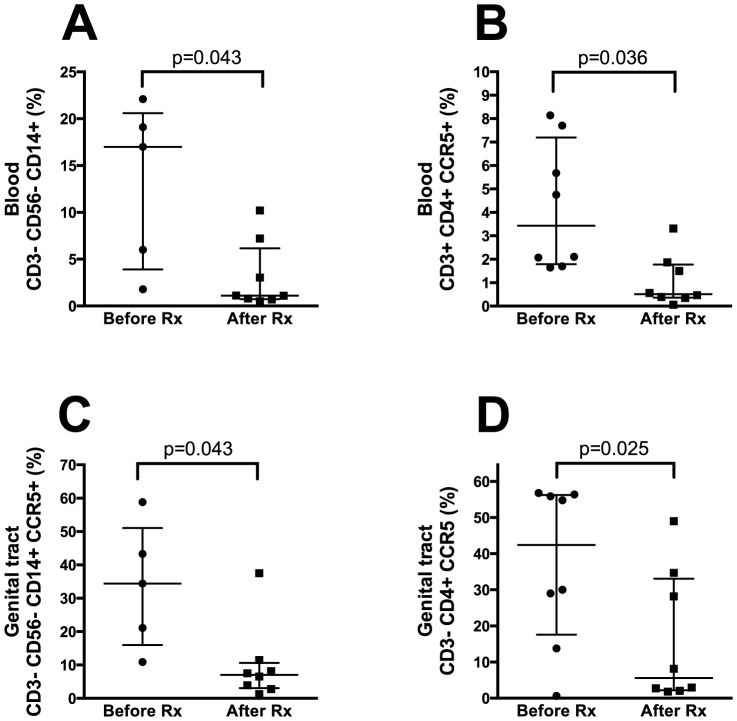
Effect of praziquantel treatment. Figures comparing FGS positive individuals in blood (A–B) and cervical samples (C–D) before and after praziquantel (40 mg/kg) treatment.

In FGS+ women treated with praziquantel, the frequency of cells in blood expressing CD14 (CD3^−^CD56^−^CD14^+^) was significantly lower after treatment (pre-treatment median 17.0% (IQR 3.9–20.6) compared to post-treatment median 1.11% (IQR 0.7–6.2), p = 0.043; Figure 5A). The level of CCR5 expression on CD4^+^ cells decreased significantly after treatment (median 3.4% (IQR 1.8–7.2) vs. 0.5% (IQR 0.4–1.8) respectively, p = 0.036; Figure 5B). However, the proportion of CD3^−^CD56^−^CD14^+^CCR5^+^ cells did not decrease significantly (p = 0.23; data not shown). The median percentage of CD4^+^ T-cells in blood was unchanged after treatment (51% before and 56% after treatment, p = 0.40; data not shown).

### Effect of praziquantel treatment on HIV target cells in genital samples

After treatment with praziquantel, the frequency of cells expressing CD14 in genital samples was significantly lower than before treatment (median 1.5% (IQR 0.8–3.6) vs. 1.0% (IQR 0.3–1.6) respectively, p = 0.043; data not shown). For CD4^+^ T-cells, the proportion was not significantly changed after treatment. The proportion of monocytes and CD4^+^ cells expressing CCR5 decreased significantly post-treatment (median 34.4% (IQR 16.0–51.1) before vs. 7.0% (IQR 3.1–10.7) after treatment, p = 0.043; Figure 5C and pre-treatment median 42.4% (IQR 17.6–56.3) vs. post-treatment median 5.6% (IQR 2.2–33.1), p = 0.025; Figure 5D).

### STI prevalence

*T. vaginalis* infection was associated with FGS (Table 1), but not associated with any of the genital cell parameters and multivariate analysis was therefore not performed. Thirty samples were available for *Chlamydia trachomatis* analysis and this was associated with CD4^+^ cells expressing CCR5 in cervical samples (p = 0.001). The other STIs were not associated with any of the studied parameters. When all STIs were added together, CD4^+^ cells expressing CCR5 in blood was associated with having at least one STI (p = 0.014), the other studied parameters were not significantly associated.

## Discussion

In this study of young women, we found that genital schistosomiasis was associated with significantly higher frequencies of CD14^+^ cells and higher frequencies of CD4^+^ cells expressing CCR5 in blood. After anti-schistosomal treatment, we found that the CD14^+^ cell population had decreased significantly in both the genital and blood compartments. Also the level of monocytes and CD4^+^ T-cells expressing CCR5 decreased in cervical cytobrush samples post treatment as well as the proportion of CD4^+^CCR5^+^ cells in blood.

Macrophages and CD4^+^ T-cells expressing the HIV co-receptor CCR5 are susceptible to HIV infection [7]. Circulating monocytes migrate to the tissues where they develop into various macrophage subpopulations [6]. Mononuclear leukocytes are interspersed in the mucosal columnar epithelium lining the endocervical channel where they may be captured by using an endocervical cytobrush [31]–[33], [35]. Vaginal macrophages are monocyte-like and express CD14 [7], [8], [36]. Since co-expression of CD14 and CD56 can occur, monocytes were defined as CD3^−^CD56^−^CD14^+^ cells [37]. Helminthic infections are known to result in a Th2-dominated immune response and *S. mansoni* induce alternatively activated macrophages that contribute to different functions, such as fibrosis, depending on the stage of the infection [38]–[40].

Women with primary or secondary syphilis, or *Herpes simplex* virus (HSV) type-1 or -2 infections, have been shown to have increased numbers of CD14 positive cells expressing CCR5 in biopsies obtained from lesions. Patients with syphilis also had increased co-receptor expression on monocytes from non-ulcerated tissue [41]. HSV-2 was associated with an increased number of activated T-cells in the genital tract [42].

Histopathological studies have shown that schistosome ova are surrounded by plasma cells, lymphocytes, eosinophil granulocytes and macrophages [20], [43]. A previous study reported high levels of CD4^+^ T-cells around calcified ova typical of chronic infection [20]. As our study found an apparent lack of effect of treatment on CD4^+^ T-cells in the genital compartment, this may indicate that there is a long-term, possibly refractory effect of schistosomal infection. The included women may already have chronic lesions if they contracted the infection at a young age. The density of macrophages, however, was higher around viable *S. haematobium* ova [20] and after treatment we found that the frequency decreased indicating that the presence of genital macrophages may be of a transient nature.

In vitro studies on peripheral blood mononuclear cells from patients with current parasitic infection indicate that their cells are more susceptible to HIV infection [44], [45]. A systemic effect of schistosomiasis was also described in individuals infected with *S. mansoni* who expressed a higher, but not statistically significant, density of the CCR5 receptor on CD4^+^ T-cells and monocytes in blood which then decreased after anti-schistosomal treatment [46]. The finding of higher levels of CCR5 expression in CD4^+^ T-cells is in accordance with our study and we also found a decrease in CCR5 expression on these cells after anti-schistosomal treatment. However, comparing schistosome infected and uninfected individuals, we did not see a higher CCR5 expression in monocytes in blood. The proportion of cells expressing CCR5, as well as the density of receptors on each cell, are likely to influence HIV susceptibility in vivo [47]–[49].

Dendritic cells are also involved in HIV transmission, possibly both by direct and indirect transmission of the virus, and may express CCR5 [50], [51]. As we did not include a marker for this cell population in our panel, we could not investigate these cells.

A crucial point is whether the cells captured by the cytobrush method are really representative for the distribution of cells deeper in the mucosa where the immunological processes take place. Another limitation to our study was the fact that samples were analyzed in two batches separated by approximately 8 months. Even though the procedures were standardized, it is possible that processing and analysis of the samples varied. We were not able to follow up any negative cases, however baseline samples of negatives were collected at both time points. We also noted that a higher number of cervical cells were found in the second round even though the same physicians collected the samples. Further, we had a limited sample size that could cause type 2 errors. We chose to use a significance level of 0.05 due to our small sample size, but because multiple parameters were tested, it is possible that some of these findings could represent false-positive results. The study should therefore be repeated in a larger sample of women. With a larger sample size, multivariate analysis would also have been possible.

The negative controls are from an area endemic for schistosomiasis and may have been exposed to the parasite. However, none of them were found to have current genital lesions. The risk of HIV infection could therefore be different for the FGS positive cases. Many contract urogenital schistosomiasis during childhood and are therefore likely to have a chronic infection, but FGS positive women may represent various stages of the disease [27]. No suitable diagnostic test is yet available for FGS [18]. Antibody tests are of limited value in endemic areas since they can not accurately differentiate between current or past disease [52] and antigen tests in blood and urine are non-specific to the site of pathology. Grainy sandy patches have been described as pathognomonic to FGS [53]. It has been indicated that genital lesions, once established, become chronic [26]. However, we cannot preclude that some negative cases have had urogenital schistosomiasis including healed genital lesions. If the negative controls had been exposed to *S. haematobium*, the impact of schistosomiasis infection on HIV target cells may be underestimated.

It has been suggested that mucosal target cell availability in the HIV negative susceptible individual may be an important factor in transmission [12], [54]. Also a higher level of CCR5 expression might increase the susceptibility to HIV infection [55]. Recruitment of HIV susceptible cells to the female genital mucosa may increase the risk of contracting the disease and could alter the progression of the infection. In addition to damaging the mucosal epithelial barrier, FGS may increase the HIV target cell population both systemically and in the mucosal compartment. Our finding that anti-schistosomal treatment decreases the fraction of HIV susceptible CD14^+^ cells and the expression of the HIV co-receptor CCR5 indicates that anti-schistosomal treatment may become an additional tool in fighting the HIV epidemic.
